# Investigation of the Effect of Occupational Noise Exposure on Blood Pressure and Heart Rate of Steel Industry Workers

**DOI:** 10.1155/2013/256060

**Published:** 2013-05-28

**Authors:** Zahra Zamanian, Reza Rostami, Jafar Hasanzadeh, Hassan Hashemi

**Affiliations:** ^1^Occupational Health Department, School of Health and Nutrition, Shiraz University of Medical Sciences, Shiraz 7153675541, Iran; ^2^Epidemiology Department, School of Health and Nutrition, Shiraz University of Medical Sciences, Shiraz, Shiraz 7153675541, Iran; ^3^Environment Research Center, Isfahan University of Medical Sciences, Isfahan, Iran

## Abstract

*Background and Objectives*. This study aimed to investigate the effect of noise exposure on blood pressure and heart rate of steel industry workers. *Materials and Methods*. In the present cross-sectional study, 50 workers were selected from a steel company in Fars province, Iran, and exposed to 85, 95, and 105 dB noise levels for 5 minutes. The participants' blood pressure and heart rate were measured using Beurer BC16 pulse meter both before and after the exposure. *Results*. The study results showed no significant difference in blood pressure and heart rate before and after the exposure. However, the workers' systolic blood pressure had increased compared to before the exposure; of course, the difference was not statistically significant (*P* > 0.05). Besides, although the subjects' heart rate had reduced in comparison to before the exposure, the difference was not statistically significant (*P* > 0.05). *Conclusion*. No significant change was observed in blood pressure and heart rate after acute exposure to 85, 95, and 105 dB noise levels.

## 1. Introduction

Noise is one of the physical factors in industries, and, today, more attention is being paid to its harmful effects. After smoking and air pollution, noise pollution is considered as the third cause of heart attack among the residents of Berlin [1]. World Health Organization (WHO) also considers noise as the third dangerous pollutant of megacities [2]. Moreover, damage to the hearing system, interference in conversation, effects on the organs of vision, effects on balance system, social disorders, psychological as well as nervous effects, impacts on electrolytes, physiological effects, and mental effects are among the noise health effects on human body [2].

Almost two thousand years ago, Pliney stated that the individuals living near noisy waterfalls tend to suffer from hearing loss sooner than other people. Also, Ramazzini reported several cases of occupational deafness in 1700 [3]. The results of a study which was conducted in 2000 showed hearing loss as the main harmful effect of long exposure to occupational noise [4]. Furthermore, Neghab et al. performed a study on the workers of petrochemical complex and revealed that noise had led to an increase in blood pressure and hearing loss in the exposed workers [5]. In the same line, Smith et al. investigated the body's physiological responses when being exposed to high-noise levels and showed that exposure to repeated, continuous noise caused the incidence of physiological as well as psychological responses and resulted in changes in heart rate as well as blood pressure [6, 7]. Moreover, according to the study by Motamedzade and Ghazaiee, exposure to higher than 85 dB noise levels increased both systolic and diastolic blood pressure, affected the working efficiency, and led to interference in conversation [8]. Ising and Michalak compared the effects of noise in both field and laboratory conditions and showed that exposure to 97 dB noise level had resulted in physiological as well as psychological changes in half of the study subjects [9].

Overall, according to the report by WHO, noise causes 4 million dollars health damage every day [10, 11], and occupational noise can put the industrial workers' health at a high risk [12].

Based on what was mentioned above and since noise can have both physiological and psychological effects on humans, the present study was conducted in order to investigate the effects of noise exposure on blood pressure and heart rate in steel industry.

## 2. Materials and Methods

The present cross-sectional study aimed to investigate the effect of occupational noise exposure on heart rate and systolic as well as diastolic blood pressure. Considering CI = 95% and power of 90%, the sample size of the study was determined as 50 subjects who were selected through simple random sampling. The inclusion criteria of the study were being physically and psychologically healthy, not smoking, not using alcohol, not taking hypnotic drugs, and not working in shifts.

The participants' demographic information was gathered through a questionnaire, and their systolic as well as diastolic blood pressure and heart rate were measured using Beurer BC16 pulse meter. In addition to assessing the workplace noise, a sample of the devices' noise was also obtained. In doing so, a microphone was attached to a worker's collar near his ear and the device's noise was recorded for 10 minutes.

Considering the features of steel industry, this study was conducted with exposure to 85, 95, and 105 dB noise levels in controlled experimental conditions for 3 consecutive days. At first, the subjects were placed in a quiet room with the background noise level of 40 dB and their systolic as well as diastolic blood pressure and heart rate was measured. Then, they were exposed to the noise recorded from the workplace at 85, 95, and 105 dB levels for 5 minutes and systolic as well as diastolic blood pressure and heart rate was measured again. In order to eliminate the sequence effect, the experiments were performed with 24 h intervals. Finally, the data were entered into the SPSS statistical software, and paired *t*-test was used in order to compare the means of the variables before and after noise exposure.

## 3. Results

The demographic characteristics of the study subjects are presented in Table 1. In this study, age, weight, height, systolic and diastolic blood pressure, and heart rate followed normal distribution.

Overall, devices were there in 38% of the workplace space. Moreover, the results of workplace noise analysis showed that, among the 60 areas under study, 13 (23%) had over 85 dB noise level and 23 (39%) had caution range noise level, that is, 65–85 dB.

Table 2 presents the subjects' systolic as well as diastolic blood pressure and heart rate before and after noise exposure in the controlled experimental conditions.

As the table depicts, the mean of systolic blood pressure has increased at all noise levels except for 85 dB; however, the changes were not statistically significant (*P* > 0.05). On the other hand, diastolic blood pressure increased a little at all the three noise levels. Besides, a decrease was observed in the mean of heart rate at all the noise levels; nevertheless, the changes were not statistically significant (*P* > 0.05).

## 4. Discussion

Up to now, the mechanism noise that affects blood pressure has not been identified. Nevertheless, several studies have shown high secretion of vasoconstrictors in urine as a result of being exposed to higher than 90 dB noise levels, which might represent the biological effects of noise exposure on blood pressure [5]. In the study by Ising and Michalak no significant difference was observed in the subjects' heart rate after acute noise exposure [9]. In general, acute exposure to 90–100 dB noise levels increases the catecholamines [5, 13] which might have led to an increase in systolic as well as diastolic blood pressure. However, the changes in blood pressure were not statistically significant, which is consistent with the findings of the present study. On the other hand, Motamedzade and Ghazaiee showed that exposure to higher than 85 dB noise levels resulted in an increase in both systolic and diastolic blood pressure, affected the working efficiency, and led to interference in conversation [8], which is in line with the results of the current study. In the study by Neghab et al., a significant difference was found between the exposed and the reference groups' mean blood pressure, which might be due to the industry under investigation as well as the study design. Moreover, the exposed and the reference groups were similar in the present study, while Neghab et al. selected two separate groups (control and exposed) in order to investigate the effects of noise. 

Overall, since blood pressure and heart rate might be affected by various factors, the results obtained regarding the effects of noise exposure on these parameters must be interpreted with caution.

## 5. Conclusion

The findings of the present study were in agreement with those of the previous studies conducted on the issue. According to the results, no significant difference was observed in blood pressure and heart rate before and after acute exposure to 85, 95, and 105 dB noise levels.

## Figures and Tables

**Table 1:** Demographic and occupational characteristics of the study subjects [mean (SD) and range].

Variable	Mean (SD)	Range	Number = *n*
Height (cm)	174 (6.14)	160–189	50
Weight (kg)	71.78 (12.16)	49–110	50
BMI	23.42 (3.28)	16.95–32.14	50
Age (years)	28.92 (5.40)	19–42	50

**Table 2:** Systolic and diastolic blood pressure and heart rate before and after the exposure to different noise levels.

Noise level	Variable	Mean (SD) before exposure	Mean (SD) after exposure	*P*-value*
85 dB	Systolic blood pressure (mmHg)	117.38 (12.32)	115.75 (11.09)	0.564
	Diastolic blood pressure (mmHg)	73.87 (10.78)	74.12 (8.69)	0.925
	Heart rate (bpm)	74.5 (8.33)	71.5 (13.06)	0.200

95 dB	Systolic blood pressure (mmHg)	111.88 (7.52)	112.12 (5.81)	0.889
	Diastolic blood pressure (mmHg)	69.37 (4.68)	69.75 (4.97)	0.623
	Heart rate (bpm)	74.5 (9.85)	71.75 (9.43)	0.147

105 dB	Systolic blood pressure (mmHg)	109.37 (12.51)	111.87 (9.81)	0.445
	Diastolic blood pressure (mmHg)	69 (6.5)	71.5 (6.54)	0.308
	Heart rate (bpm)	71.12 (9.26)	70.87 (11.03)	0.895

*Paired *t*-test.
